# Experiences with archived raw diffraction images data: capturing cisplatin after chemical conversion of carboplatin in high salt conditions for a protein crystal

**DOI:** 10.1107/S0909049513020724

**Published:** 2013-10-01

**Authors:** Simon W. M. Tanley, Kay Diederichs, Loes M. J. Kroon-Batenburg, Antoine M. M. Schreurs, John R. Helliwell

**Affiliations:** a School of Chemistry, Faculty of Engineering and Physical Sciences, University of Manchester, Brunswick Street, Manchester M13 9Pl, UK; b Department of Biology, University of Konstanz, Germany; c Crystal and Structural Chemistry, Bijvoet Center for Biomolecular Research, Faculty of Science, Utrecht University, Padualaan 8, 3584 CH Utrecht, The Netherlands

**Keywords:** cisplatin, carboplatin, conversion, archiving, raw diffraction images data

## Abstract

The archiving of raw diffraction images data is the focus of an IUCr Diffraction Data Deposition Working Group (see http://forums.iucr.org/). Experience in archiving and sharing of raw diffraction images data in collaboration between Manchester and Utrecht Universities, studying the binding of the important anti-cancer agents, cisplatin and carboplatin to histidine in a protein, has recently been published. Subsequently, these studies have been expanded due to further analyses of each data set of raw diffraction images using the diffraction data processing program *XDS*. The raw diffraction images, measured at Manchester University, are available for download at Utrecht University and now also mirrored at the Tardis Raw Diffraction Data Archive in Australia. Thus a direct comparison of processed diffraction and derived protein model data from *XDS* with the published results has been made. The issue of conversion of carboplatin to cisplatin under a high chloride salt concentration has been taken up and a detailed crystallographic assessment is provided. Overall, these new structural chemistry research results are presented followed by a short summary of developing raw data archiving policy and practicalities as well as documenting the challenge of making appropriate and detailed recording of the metadata for crystallography.

## Abstract 2

Archiving of raw diffraction images data has led to new structural chemistry information being obtained for previously published results, which leads to the conclusion that carboplatin has partially converted to cisplatin in the high NaCl concentration conditions used in the crystallization procedure.

## 1. Introduction   

The archiving of raw diffraction images data is the focus of an IUCr Diffraction Data Deposition Working Group (see http://forums.iucr.org/). Experience in archiving and sharing of diffraction data in collaboration between Manchester and Utrecht Universities studying the binding of the important anti-cancer agents, cisplatin and carboplatin to histidine in a protein has recently been described in detail (Tanley *et al.*, 2103*a*). Subsequently, these studies have been expanded when one of us (KD) joined in the detailed further analyses of each set of raw diffraction images with *XDS* (Kabsch, 1988 ▶). The motivation for this collaboration was due to the title of the previously published article (Tanley *et al.*, 2013*a* ▶) which suggested that a rather comprehensive approach was taken to compare software packages, with the implicit intention to correlate some metrics of data quality [*R*_merge_, *I*/σ, *CC*_1/2_, completeness and diffraction precision index (DPI) values, all as a function of resolution] with the amount of biological insight that these data allow one to obtain. Thus, a comprehensive investigation becomes even more useful with every addition that broadens its basis, which suggested contributing the processing with yet another widely used software package (*XDS*) on the existing raw diffraction data images. All of the raw diffraction images data can be found at http://rawdata.chem.uu.nl/#0001, except for 4g4c, which are at http://rawdata.chem.uu.nl/#0002, and now also mirrored at the Tardis Raw Data Archive in Australia (http://vera183.its.monash.edu.au/experiment/view/40/). The raw data sets, being a part of the supplementary materials available with the previously published article (Tanley *et al.*, 2013*a* ▶), have also led to a Supplementary Materials button being added to the *Journal of Applied Crystallography* contents page for our article (doi:10.1107/S0021889812044172), the first of its kind. The raw diffraction data were measured on home X-ray sources at Manchester University but our study has implications for synchrotron radiation facilities, where a large fraction of macromolecular X-ray data these days are measured. That said, the lead investigator for any given synchrotron radiation beam time proposal presumably would hold the data management responsibilities of their relevant funding organization and would thus finally place the data for any given publication with their preferred raw data archive in addition to the formally required Protein Data Bank (PDB) depositions of processed and derived data. It is noted that various Universities in the UK are setting up research data archives for use by its research staff, and to serve all research fields, so as to satisfy the existing, and increasing, mandates of funding organizations. Specialist needs such as at CERN are being assumed by Universities as to be met centrally, *i.e* at CERN itself. Neutron facilities are also in parallel setting up or have set up raw data archives hosted by the neutron facility itself. Synchrotron radiation facilities have a variety of ‘raw data retention policies’ (see the IUCr Forum for details).

This study focuses on a direct comparison of processed diffraction and derived protein model data from *XDS* (Kabsch, 1988 ▶) with the results published previously (Tanley *et al.*, 2013*a* ▶). The possible issue of partial conversion of carboplatin to cisplatin under a high chloride salt concentration (Tanley *et al.*, 2012*a* ▶; Gust & Schnurr, 1999 ▶) has been taken up based on new evidence coming from the re-processing of our diffraction images as well as re-visiting the already published data and a detailed crystallographic assessment is provided.

Overall we give a mix of research results in a particular study (anti-cancer agents in their chemical behaviours and their binding to histidine in a protein) and also a short summary of developing raw data archives for diffraction data images in terms of policies and practicalities. We will also document the not insignificant challenge of making appropriate and detailed recording of the metadata for crystallography raw diffraction data.

## 2. Methods   

Details of all the crystallization conditions and the X-ray data collection strategy for each crystal has already been previously documented (Tanley *et al.*, 2012*a* ▶,*b* ▶, 2013*a* ▶). PDB IDs: 4dd0, 4dd1, 4dd2, 4dd3, 4dd4, 4dd6, 4dd7, 4dd9, 4dda, 4ddb, 4ddc, 3txb, 3txd, 3txe, 3txi, 3txj, 3txk, 3txf, 3txg, 3txh, 4g49, 4g4a, 4g4b, 4g4c, 4g4h.

Graphs of the anomalous signal-to-noise ratio per resolution shell for each crystal processed by the different software packages are given in the supplementary materials.^1^

## 3. Results   

### 3.1. Anti-cancer agents in their chemical behaviours and their binding to histidine in a protein   

**Figure 1:**
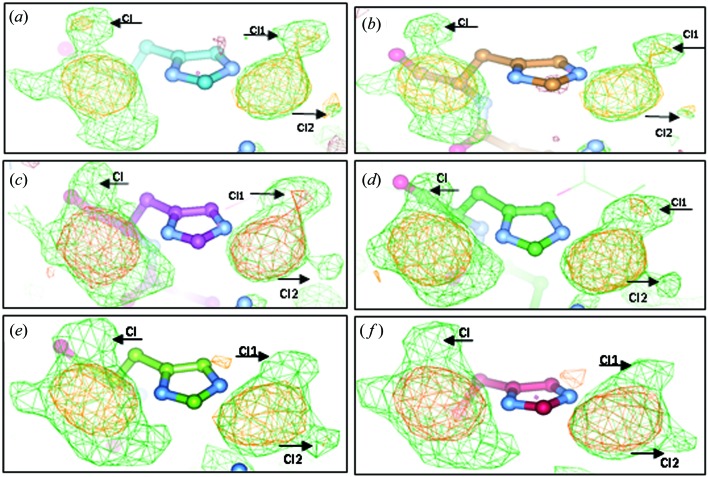
The two binding sites on the His-15 residue of HEWL. *F*_o_ − *F*_c_ density OMIT maps (green) and anomalous difference density (orange) maps at the 3σ cut-off level for (*a*) and (*b*) 4dd7 processed by *XDS* and *EVAL*, (*c*) and (*d*) 4dd9 processed by *XDS* and *EVAL* and (*e*) and (*f*) 4g4c processed by *XDS* and *EVAL*, respectively. The Nδ binding site is on the left-hand side and the N∊ binding site is on the right-hand side. The arrows show where the possible Cl atom locations are based on anomalous difference density being seen.

From previous studies (Tanley *et al.*, 2012*a* ▶,*b* ▶) it is shown that carboplatin bound to the Nδ and N∊ atoms of His-15 of HEWL in DMSO crystallization conditions. It was also shown that cisplatin did likewise but in addition it proved possible to show that its binding also took place over prolonged chemical exposure of around a year; carboplatin-based lysozyme crystals under aqueous conditions produced crystals too fragile after a year’s storage to study successfully. Through the Utrecht archive, one of us (KD) downloaded the data sets diffraction images reanalysed here. Thus, using *XDS* to process these archived raw diffraction images (PDB ID 4dd7/4dd9/4g4c), clearly significant anomalous difference density peaks in the carboplatin binding site were seen; appropriately placed for where a Cl atom in cisplatin would be expected to be for the Nδ binding site and for two Cl atoms in the N∊ binding site. Comparing these anomalous difference density peak heights with the peak heights from the *EVAL* (Schreurs *et al.*, 2010 ▶), *MOSFLM* (Leslie, 1999 ▶) and PROTEUM/D*Trek (Bruker, 2006 ▶; Pflugrath, 1999 ▶) processing programs (Tanley *et al.*, 2013*a* ▶) (Table 1 ▶), it was noted that for 4dd7, these three Cl atoms were mis-interpreted first of all by us (ST and JRH), with evidence of their presence coming from all four processing programs. Whereas, for 4dd9 and 4g4c, there is no evidence for all of these Cl atoms above our 3σ cut-off level, with only one Cl in the N∊ binding site being present above the 3σ cut-off level. This new finding, along with re-modelling the *EVAL* deposited data, leads to the conclusion that the carboplatin could in fact have been partially or possibly even fully converted to cisplatin. This chemical conversion and the respective percentages were then evaluated for each binding site (Fig. 1 ▶). The partial transformation of carboplatin to cisplatin has been seen previously reported on in solution (Gust & Schnurr, 1999 ▶). In a 0.9% NaCl solution, 10% of the carboplatin had converted to cisplatin after 28 d storage at room temperature and the concentration of NaCl used in our crystallization conditions (10%) and the storage of our crystals between days and months at room temperature (Tanley *et al.*, 2012*a* ▶,*b* ▶) could indeed facilitate this partial conversion. The largest transformation took place in 4dd7, a crystal with only three days of storage. *OVERLAPMAP* (CCP4 package) was used to give the statistical correlation coefficient of which ligand could be bound at each binding site based on the 2*F*_o_ − *F*_c_ maps as well as *SHELX* (Sheldrick, 2008 ▶) being used to obtain the refined occupancies of the Pt atom centre at each binding site, and the occupancy values of each bound ligand remaining atoms at each binding site. This information, using both *EVAL* and *XDS* processing programs for each dataset (4dd7, 4dd9 and 4g4c), is given in Tables 2 ▶ and 3 ▶.

## 4. Discussion   

### 4.1. Anti-cancer agents in their chemical behaviours and their binding to histidine in a protein   

Archiving our raw diffraction images enabled us to re-process them, resulting in biological insight going beyond that of our previously published results (Tanley *et al.*, 2013*a* ▶; http://rawdata.chem.uu.nl/#0001; http://vera183.its.monash.edu.au/experiment/view/40/). It is now thought that instead of carboplatin binding alone to HEWL in DMSO media (Tanley *et al.*, 2012*a* ▶,*b* ▶) we actually see a mixture of carboplatin and cisplatin in the binding sites due to the partial conversion of carboplatin to cisplatin in the high salt conditions (10% NaCl) used for the crystallization procedure. Table 1 ▶ shows the anomalous difference density peak heights in the binding sites, attributed to Cl atoms for different software programs. These confirm that, for 4dd7, anomalous difference density for these Cl atoms are seen and should have been noted previously and, also, this dataset shows the most conversion of carboplatin to cisplatin based on the correlation coefficients out of *OVERLAPMAP* (Table 2 ▶). However, we had set up clear chemical conditions and carboplatin only naturally was what we, wrongly as it turns out, expected. Whereas for 4dd9 and 4g4c, the anomalous difference density peaks are weaker, and thus harder to determine that the Cl atoms are in fact present. From the anomalous difference density peak heights, along with correlation coefficient of cisplatin/carboplatin binding to the 2*F*_o_ − *F*_c_ maps in *OVERLAPMAP* as well as the refined occupancy values from *SHELX* (Tables 1 ▶–3 ▶ ▶), it can be noted that carboplatin has been partially converted to cisplatin in the high salt conditions used for the crystallization procedure. Also, the correlation coefficient values from Table 2 ▶ for each ligand as well as the occupancy values for each ligand in Table 3 ▶ generally correlate very well between the *XDS* and *EVAL* refined models.

Based on the new finding that carboplatin has partially converted to cisplatin in the high NaCl salt content used for the crystallization conditions, a new study, involving crystallization of HEWL with carboplatin in non-NaCl conditions, is underway (Tanley *et al.*, 2013*b* ▶).

### 4.2. Developing raw data archiving policies and practicalities for diffraction data images and the challenges of making metadata available for crystallography   

Archiving of raw diffraction data images policy and practicalities for crystallography is at its early stages (see the IUCr Forum documents); very reasonably the issues involve costs *versus* benefits but within a fairly rapid expansion of technical options such as University archives becoming established and cloud storage commercial options becoming more commonly available (Helliwell *et al.*, 2012 ▶). In Tanley *et al.* (2013*a* ▶) the very pragmatic method of using a personal web link held at Utrecht University for our various raw data sets was used. From the results shown here, without this web link archiving, we would not have re-examined the previous diffraction data (Tanley *et al.*, 2012*a* ▶,*b* ▶, 2013*a* ▶) and thus would have missed this most interesting chemical effect of partial conversion of carboplatin to cisplatin.

Providing sufficiently rich metadata to fully describe raw diffraction data is important for carrying out data processing of diffraction images with subsequent software. One very specific point seen in this study was that *XDS* required manual input of the rotation axis direction and the direction of the detector *X*/*Y* axes for Bruker CCD diffractometer data, whereas, with the *EVAL* software, they were readily interpreted from the header information of the diffraction images. Our previously published work (Tanley *et al.*, 2013*a* ▶) presented a detailed and rich description of metadata for two commercial area detector diffractometers and is thus, we hope, an exemplar of what detail is required.

## 5. Conclusions   

The studies outlined here focused on the advantages of archiving the raw diffraction images for X-ray crystallography, *via* a relatively simple personal web link method rather than, say, a formal single University or centralized raw data archive. Due to archiving our raw diffraction images data, new findings have been achieved owing to re-processing the images with *XDS* as well as re-examining the previously published data. Thus, we now see carboplatin partially converting to cisplatin in the crystal structure due to the high NaCl salt concentrations used in the crystallization conditions. This has then led us to try to find new crystallization conditions without NaCl to seek to capture carboplatin binding on its own.

## Supplementary Material

Supplementary figure 1. Anomalous signal-to-noise ratio for each crystal processed with the different software packages. DOI: 10.1107/S0909049513020724/ys5072sup1.pdf

## Figures and Tables

**Figure 2:**
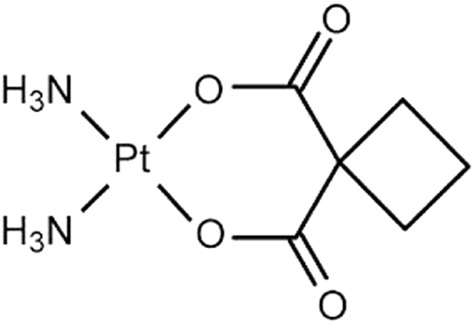
Chemical structure of carboplatin. The carboplatin moiety used differs for each structure based on the density seen at each binding site.

**Table 1:** Anomalous difference density peak heights ( cut-off level) in the N and N binding sites for Cl atoms for three different datasets using a number of different processing programs

		Anomalous peak height		
		N Cl	N Cl1	N Cl2
HEWL co-crystallized with carboplatin in 10% NaCl and DMSO media with glycerol as the cryoprotectant	3txh *PROTEUM2*	4.4	4.7	4.4
	*MOSFLM*	3.6	4.8	4.2
	4dd7_*EVAL*	3.6	3.6	3.6
	*XDS*	3.9	4.0	3.8
HEWL co-crystallized with carboplatin in 10% NaCl and DMSO media with paratone as the cryoprotectant	3txi d*TREK	0	0	0
	*MOSFLM*	2.5	4.2	2.2
	4dd9_*EVAL*	3.0	3.8	0
	*XDS*	2.8	4.1	2.4
HEWL co-crystallized with carboplatin in 10% NaCl and DMSO media studied at room temperature	4g4c_*EVAL*	2.4	2.5	2.8
	*PROTEUM2*	0	0	0
	*XDS*	0	3.1	0

**Table 2:** Correlation coefficient of cisplatin or carboplatin binding in the 2*F*_o_ *F*_c_ map for both the N and N atoms of His-15 using *OVERLAPMAP* in *CCP4i*, with the Pt centre removed, *i.e* the calculation is just for the remaining bound ligands Note that in this calculation each ligand is considered separately whereas in the *SHELX* calculation summarized in Table 3 ▶ both of the ligand occupancies are refined in the same calculation.

		N binding site		N binding site	
		Carboplatin	Cisplatin	Carboplatin	Cisplatin
HEWL co-crystallized with carboplatin in 10% NaCl and DMSO media with glycerol as the cryoprotectant	4dd7_*EVAL*	0.09	0.22	0.28	0.24
	*XDS*	0.12	0.36	0.54	0.02
HEWL co-crystallized with carboplatin in 10% NaCl and DMSO media with paratone as the cryoprotectant	4dd9_*EVAL*	0.64	0.24	0.21	0.00
	*XDS*	0.60	0.32	0.25	0.11
HEWL co-crystallized with carboplatin in 10% NaCl and DMSO media studied at room temperature	4g4c_*EVAL*	0.22	0.33	0.42	0.16
	*XDS*	0.21	0.32	0.30	0.05

**Table 3:** Refined occupancy values obtained using *SHELX* (Sheldrick, 2008 ▶) of the Pt atom centre at each binding site refined separately as well as the occupancies of the remaining bound ligand atoms at each binding site; these occupancies were obtained with *SHELX* having both ligands being considered in the refinement simultaneously

	N binding site			N binding site		
	Pt	2Cl/1N	Carbopt moiety†	Pt	2Cl‡	Carbopt moiety†
4dd7_*EVAL*	75	48	22	55	26	24
*XDS*	65	29	41	55	10	40
4dd9_*EVAL*	70	62	8	48	3	47
*XDS*	65	41	29	46	28	22
4g4c_*EVAL*	83	38	32	48	7	43
*XDS*	80	33	37	49	8	42

† See Fig. 2 ▶.

‡ The N binding site only contains two atoms bound to the Pt centre, unlike the N binding site where three atoms are seen bound to the Pt centre.

## Footnotes

1 Supplementary data for this paper are available from the IUCr electronic archives (Reference: YS5072). Services for accessing these data are described at the back of the journal.
