# Supplementary material for: Experiences with archived raw diffraction images data: capturing cisplatin after chemical conversion of carboplatin in high salt conditions for a protein crystal
Source: J Synchrotron Radiat. 2013 Oct 1;20(Pt 6):880–3. doi: 10.1107/S0909049513020724 (PMC3795548; doi:10.1107/S0909049513020724)

**Supplementary Materials**

**Figure S1** Anomalous signal to noise ratio for each crystal processed with the different software packages.

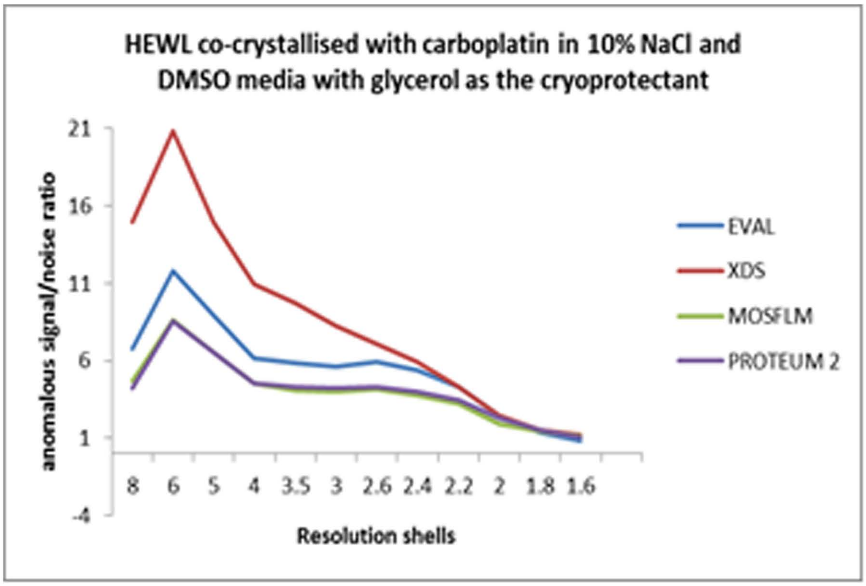


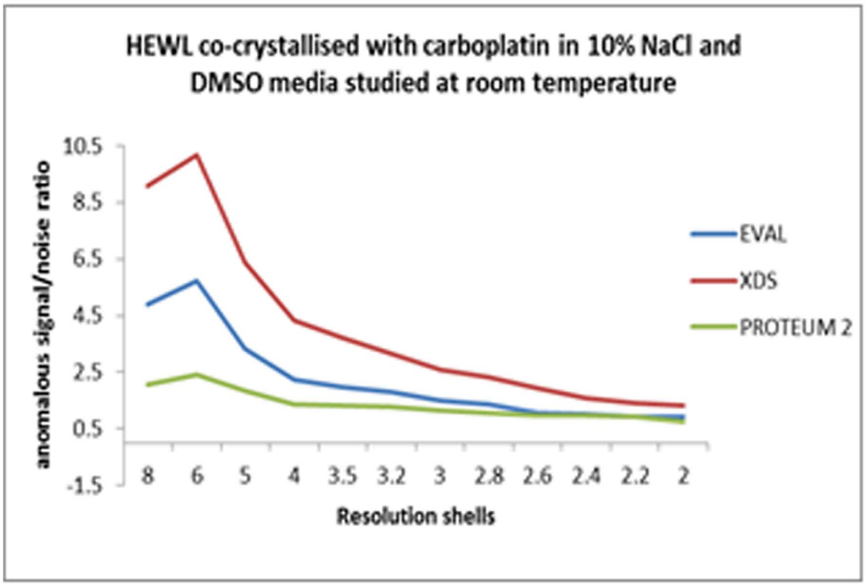


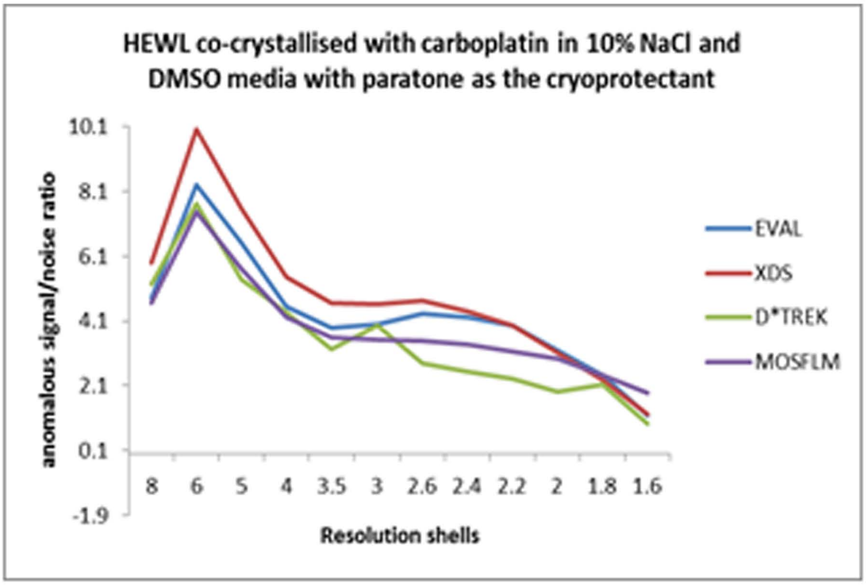

Supplement: Supplementary file 1 [file s-20-00880-sup1.pdf]
